# “Going with the Flow” or Not: Evidence of Positive Rheotaxis in Oceanic Juvenile Loggerhead Turtles (*Caretta caretta*) in the South Pacific Ocean Using Satellite Tags and Ocean Circulation Data

**DOI:** 10.1371/journal.pone.0103701

**Published:** 2014-08-06

**Authors:** Donald R. Kobayashi, Richard Farman, Jeffrey J. Polovina, Denise M. Parker, Marc Rice, George H. Balazs

**Affiliations:** 1 Pacific Islands Fisheries Science Center, National Marine Fisheries Service, National Oceanic and Atmospheric Administration, Honolulu, Hawaii, United States of America; 2 Aquarium des Lagons, Nouvelle-Calédonie; 3 Joint Institute for Marine and Atmospheric Research, University of Hawaii, Newport, Oregon, United States of America; 4 Hawaii Preparatory Academy, Kamuela, Hawaii, United States of America; Lund University, Sweden

## Abstract

The movement of juvenile loggerhead turtles (n = 42) out-fitted with satellite tags and released in oceanic waters off New Caledonia was examined and compared with ocean circulation data. Merging of the daily turtle movement data with drifter buoy movements, OSCAR (Ocean Surface Current Analyses - Real time) circulation data, and three different vertical strata (0–5 m, 0–40 m, 0–100 m) of HYCOM (HYbrid Coordinate Ocean Model) circulation data indicated the turtles were swimming against the prevailing current in a statistically significant pattern. This was not an artifact of prevailing directions of current and swimming, nor was it an artifact of frictional slippage. Generalized additive modeling was used to decompose the pattern of swimming into spatial and temporal components. The findings are indicative of a positive rheotaxis whereby an organism is able to detect the current flow and orient itself to swim into the current flow direction or otherwise slow down its movement. Potential mechanisms for the means and adaptive significance of rheotaxis in oceanic juvenile loggerhead turtles are discussed.

## Introduction

Loggerhead turtles (*Caretta caretta*) are a circumglobal species of sea turtle found throughout temperate and tropical oceans and seas [1]. Loggerhead turtle nesting is restricted to a few locations in major ocean basins such as the Southeastern USA, the Mediterranean, Oman, Western Australia, and Southern Japan, where females lay their eggs on sandy beaches [2], [3]. After hatchlings leave the nesting beaches, they are thought to remain oceanic for 7–12 years before migrating to coastal habitats [4]. Despite good swimming abilities, juvenile loggerhead turtles are thought to drift passively for a significant portion of their existence on the high seas [5]. In contrast, Polovina et al. [6] found that active swimming seemed to be a large component of overall movement for oceanic juveniles. It is likely that a behavioral response cued by current flow orientation (i.e., rheotaxis) is involved. With passive drifting, active swimming, and rheotactic responses, it is important to know the magnitude and direction of ocean currents the oceanic juveniles have to contend with, as it is clear that the response of an organism to a moving flow field can be highly complex [7]. For oceanic juvenile loggerhead turtles, a key issue is their placement in the vertical portion of the water column, which will be closely linked to their diving behavior.

Oceanic juvenile diving behavior is not well studied, with scant data from only a small number of tagged individuals. Studies in the Atlantic have shown that oceanic juveniles spend ∼75% of their time in the upper 5 m of the water column [4]. Studies in the Pacific have indicated oceanic juveniles may spend more time in deeper layers. For example, Polovina et al. [8], and Polovina et al. [9] found that oceanic juveniles spend ∼90% of their time in the upper 40 m of the water column, with 75% of the time spent in the upper 15 m, and 40% of the time in the upper 1 m. Howell et al. [10] found that oceanic juveniles spend ∼90% of their time in the upper 15 m and 80% of their time in the upper 5 m of the water column. While loggerhead turtles can dive to much deeper depths, it is clear oceanic juveniles primarily use the upper layers (5–40 m) of the water column where the wind-driven component may be more important than large scale (e.g., geostrophic) water movements. Although studies have attempted to compare oceanic juvenile movement to ocean currents, these approaches often use geostrophic currents, which may not be appropriate for this situation. Alternative approaches using field measurements or high-resolution ocean circulation models are needed, with careful attention to matching the turtle data to the appropriate vertical layers of current flow. Combining turtle movement data with depth appropriate ocean circulation models will aid in our understanding of rheotaxis in juvenile turtle ecology.

Rheotaxis in the aquatic environment is defined as the response orientation of an organism to a current flow. The rheotaxis can either be positive (turning to face into the current) or negative (turning to face away from the current). The mechanism involved in the rheotaxis can be active (e.g., turning caused by behavior) or passive (e.g., turning caused by body shape hydrodynamics). Rheotaxis is thought to occur throughout the animal kingdom and has been suggested for organisms as different as spermatozoa [11], tadpoles [12], and whale sharks [13]. Many occurrences have been documented from laboratory studies, but the experimental apparatus often confounds such studies since multiple cues may be available to the organism making it difficult to isolate pure rheotaxis. Pure rheotaxis occurs when the organism is responding only to the current flow and not following any chemical signal or similar fluid-borne plume. Often, orientation to a current is facilitated artificially by the laboratory apparatus via visual or tactile feedback to the container or substrate, which may or may not mimic natural conditions for the organism under study. Field studies with oceanic organisms likely offer the best opportunity to document true rheotaxis whereby the primary cue available to the organism is the current flow with few other easily sensed cues available to assist in orientation.

Vestibular senses such as the inner ear of vertebrates and statoliths of invertebrates, as well as the mechanoreceptory lateral line of fishes and amphibians, are likely useful for detecting bodily orientation with respect to a current flow. In addition to the normal suite of senses (hearing, sight, touch, smell, taste), it is worth noting that loggerheads, like all vertebrates, have vestibular sensory organs in the inner ear [14] for detecting motion. While this sense is generally useful for equilibrium and coordinated movement, the vestibular sensory organs are thought to also be used by sea turtles for subsurface orientation to wave direction based on the orbital movements of the water parcel within the wave swell [15]. Additionally, loggerheads have been well demonstrated to have magnetic orientation abilities [16].

To investigate the role of rheotaxis, an analysis of movement dynamics was undertaken using satellite tag data of oceanic juvenile loggerhead turtles in the South Pacific and currents flows from a variety of sources. This tagging experiment was not initially targeted towards a rheotaxis experiment but was instead an exploratory tagging experiment to characterize range of juvenile loggerhead turtle movement in the South Pacific and to ascertain presence of any potential “hot spots” [17], [18]. However, the data are amenable for an ex post facto investigation of rheotaxis, when compared to a suite of prevailing flow fields for the region. These current flow sources include actual field measurements (NOAA, National Oceanic and Atmospheric Administration, drifter buoys), satellite remotely sensed estimates (NOAA OSCAR), and modeled estimates (HYCOM). Where possible, vertical layering of current flow was examined in conjunction with the tagged animal movements.

## Materials and Methods

### Satellite Track Data

Juvenile loggerhead turtles (n = 42) were satellite tagged and released simultaneously off the coast of New Caledonia at 170.86 E longitude, 29.803 S latitude on 9 September 2008. The point of release was in international waters approximately 205 nm southeast of Norfolk Island and was conducted from a French naval vessel. All turtles were captive reared at the Aquarium des Lagons (Aquarium of the Lagoons) in Noumea, New Caledonia from hatchlings collected from multiple excavated beach nests in New Caledonia. The turtles were 1 year and 7 months old at the time of release. Their lengths varied from 24.0 to 34.3 cm straight carapace length (median = 28.1 cm) and their weights varied from 3.2 kg to 8.1 kg (median = 4.6 kg) upon release. The turtles were tracked using Wildlife Computers Smart Position or Temperature (SPOT5) satellite transmitting tags. The SPOT5 tags were affixed to the turtle carapace using epoxy resin and fiberglass cloth [19]. These tags, like many other satellite tags, utilize the Argos satellite network for geopositioning. The tags used in this experiment were programmed with a 6/42 transmission duty cycle (6 hours on, 42 hours off per 48 hour interval) to conserve battery output. All applicable government rules for proper sea turtle care and humane treatment were followed (no animals were sacrificed), and permit was obtained from the New Caledonia government for the conduction of this experiment (permit 99–209 issued 19 March 1999). The raw position data from the satellite tags were processed with Bayesian State Space Modeling (SSM) using published approaches [20], [21] coded in the statistical programming languages R and WinBUGS. The SSM procedure takes into account the Argos Location Classes embedded in the raw track data reflecting the degree of accuracy of the calculated position and the spatio-temporal patterning in the raw track data to statistically produce the most likely daily tracks from the irregularly spaced input data. Summary statistics were performed upon the 42 SSM tracks upon completion of the experiment, which was deemed concluded when the last satellite tag ceased transmission on 24 August 2009 after 350 days of transmission. These daily satellite track data were matched using a spatio-temporal query procedure to computationally align the track positional data to several estimates of current flow, an approach similar to previous methodologies used to interpret pelagic movement dynamics [17], [22] and is further described below.

### NOAA Drifter Buoy Data

The drifter buoy dataset used in this study is from the NOAA Global Drifter Program, which consolidates a variety of international efforts deploying surface drifter buoys. These devices consist of a small surface buoy with a subsurface drogue (sea anchor) attached by a tether line; a transmitter on the buoy sends the positional and other data to satellites. The drogue comprises most of the surface area of the instrument and is centered at a depth of 15 m below the sea surface. Positions are transmitted 16–20 times per day, and these are used to provide standardized and interpolated ¼ day location data. At any given time, the program uses around a thousand active buoys to maintain a global 5×5 degree latitude and longitude coverage of the world oceans.

Drifter buoy data was downloaded for the time period September 2008 to August 2009. Daily current vectors were averaged at a resolution of 1 degree latitude and longitude from this drifter buoy data coverage, and these average current vectors were used to populate daily grids. These grids were queried for spatial-temporal matching to the turtle track data.

### NOAA OSCAR Data

The NOAA Ocean Surface Current Analyses - Real time (OSCAR) project uses satellite altimeter data to estimate sea level height and satellite scatterometer data to estimate wind speed and direction. The merging of these remotely sensed data products produces an estimate of mixed layer ocean circulation. This flow is driven by both the geostrophic component and the wind component, with the final product being tuned to represent the movement of a drogue at 15 m depth, i.e., the NOAA drifter buoys mentioned in previous section.

OSCAR data covering the temporal domain of September 2008 through August 2009 (5-day mean) and the spatial domain of 120E to 140W longitude and 0 to 60S latitude were downloaded from the OSCAR data portal website. NetCDF data files were extracted to ascii using routines coded in the statistical programming language R, and the data were gridded to a uniform 0.3333 degree latitude and longitude grid for every 5-day mean. These grids were queried for spatial-temporal matching to the turtle track data.

### HYCOM Data

The HYbrid Coordinate Ocean Model (HYCOM) is a data-assimilative ocean circulation model developed by the multi-institution HYCOM Consortium and was the source of the modeled ocean circulation flow fields used in this study. Forcing and assimilation for HYCOM are accomplished using the Navy Operational Global Atmospheric Prediction System (NOGAPS) winds to drive the dynamics, and the Navy Coupled Ocean Data Assimilation (NCODA) system to incorporate external measurements of satellite altimetry, sea surface temperature, and *in-situ* vertical profiles of temperature and salinity. The NOGAPS and NCODA data can vary in temporal resolution from hours to weeks as well as be snapshot inputs from vessels, moorings, or profiling buoys; however, the standard temporal resolution of the HYCOM output data is daily, which was used in this study. This daily HYCOM data is a subset of the University of Miami global model using K-Profile Parameterization for characterizing the mixed layer and is made available at the Asia-Pacific Data-Research Center (APDRC) at the International Pacific Research Center (IPRC), School of Ocean and Earth Science and Technology, University of Hawaii (point of contact Dr. Yanli Jia). Daily HYCOM data covering the temporal domain of September 2008 through August 2009 and the spatial domain of 120E to 140W longitude and 0 to 60S latitude were downloaded using the APDRC/IPRC website as a data conduit. NetCDF data files were extracted, gridded and/or averaged, as described below, to ASCII data files using routines coded in the statistical programming language R.

HYCOM is vertically structured with 33 layers ranging in thickness from 5–500 m, with thinner layers in the upper portions of the water column and an average layer width of 10 m in the upper 40 m of the water column. For this exercise, 3 different vertical strata of the HYCOM data were used. In the first application, only the uppermost layer representing water motion in the upper 5 m was extracted and used for matching with the satellite track data (henceforth termed “shallow”). In the second application, the uppermost 4 HYCOM layers were averaged using the R language routines and utilized for matching with the satellite track data. This 4-layer average represents water motion in the upper 40 m of the water column (henceforth termed “intermediate”). In the third application, the uppermost 7 HYCOM layers were averaged using the R language routines and utilized for matching with the satellite track data. This 7-layer average represents water motion in the upper 100 m of the water column (henceforth termed “deep”). The horizontal structure of the HYCOM is a variable width resolution averaging ∼1/12^th^ (∼0.0833) degree over both longitude and latitude. For ease of use, the daily variable resolution output was regridded to a daily uniform 0.1 degree coordinate system in latitude and longitude using the mapping software Generic Mapping tools (GMT) subroutines named *blockmean* and *surface* [23]. These grids were queried for spatial-temporal matching to the turtle track data.

Quarterly average current fields were created and plotted with GMT coinciding with the Austral (southern hemisphere) climate pattern (spring = September–November, summer = December–February, autumn = March–May, and winter = June–August) to examine large-scale oceanographic features.

### Merging of Tracks and Currents

The daily SSM satellite track data was matched in time and space to the following estimates of current flow: 1) the daily 1 degree resolution drifter grids, 2) the 5-day 0.3333 degree resolution OSCAR grids, 3) the daily 0.1 degree resolution HYCOM shallow grids, 4) the daily 0.1 degree resolution HYCOM intermediate grids, and 5) the daily 0.1 degree resolution HYCOM deep grids. For each instance, the track data were matched to a particular ocean current u-component (east/west) and v-component (north/south), henceforth simply referred to as “u” and “v”, respectively, using a computer program written in TrueBasic. In this step, the nearest pixel of ocean current information was matched to the daily location in the SSM track data using large multidimensional matrices indexed in the specified resolutions over time and space. These u and v were used to estimate the drift portion of the daily track displacement and to infer the swimming vector on a daily basis using simple vector geometry. In other words, for each daily time step, there was an overall resultant tag displacement as measured from the adjacent SSM track data locations. The ocean current u and v at the starting location were used to specify the predicted drift displacement. Subtraction of this drift vector from the resultant vector yields the swimming vector needed to explain the tag displacement under those particular ocean current conditions. These 3 daily vectors of current, tag displacement, and turtle swimming were tabulated into polar frequency histograms using the GMT software and examined in aggregate. The difference between the swimming vector and the current vector was also examined after conversion to a polar coordinate system where the relative swimming orientation with respect to the current direction could be identified, irrespective of their absolute directions. This latter measure is intended to address the existence of rheotaxis.

Due to the complex nature of the data structure (e.g., repeated measurements on a tagged individual and possible issues with pseudoreplication coupled with spatial and temporal autocorrelation), statistical analysis proceeded along 2 different approaches for added robustness in testing for rheotaxis. In the first approach, conventional circular statistics were used with a simple adjustment to the degrees of freedom. Adjustments to degrees of freedom to resolve pseudoreplication issues in the test statistic have been used before. For example Hedges [24] proposes a modification to standard statistical tests using an adjustment to the degrees of freedom based on the number of “clusters” that formed the basis for a pseudoreplication issue. This is essentially adjusting the analysis to better match the “effective sample size” of the specific situation [25], [26]. In addition, the commonly used Satterthwaite Approximation [27] implements an adjustment to the denominator degrees of freedom in a statistical test based on the variance structure of the underlying model (i.e., the structure of the random effects) to determine the degrees of freedom. In a genetic analysis, this can manifest itself in using the number of mothers, for example, rather than the number of individuals in an analysis [28]. In the context of the present study, there is a clear analogy and logic to use the number of tagged individuals as the effective sample size.

In this first approach, statistical tests of the polar data were undertaken using a Rayleigh’s *z* test of directional uniformity following the circular distribution methodology in Chapters 24–25 of Zar [29]. The only adjustment to the Rayleigh’s *z* test was to use the sample size of tagged turtles (n = 42) for determining the critical value of the Rayleigh’s *z* statistic (Table B.32 in Zar), since the numerous tag observations (n = 7334) do not represent independent observations and are likely an instance of pseudoreplication [30]. An adjusted degrees of freedom approach was applied, similar to that used previously in non-parametric tests involving tagged animal data [22]. For simplicity, the original equation used to generate the Zar [29] critical values of the Rayleigh’s *z* statistic (Equation 6 from [31]) was inverted to calculate an exact probability given a particular Rayleigh’s *z* value and particular degrees of freedom (n = 42 in this instance). The purpose of the Rayleigh’s *z* tests was to determine whether a prevailing absolute or relative direction exists in the data.

In the second approach, statistical tests of the polar data were undertaken using an approach called second-order statistical analysis following the methods of Batschelet [32] and the software package Oriana (version 4). This approach is useful when multiple observations are made on a single individual, and hence the independence of observations becomes questionable. In this situation it then becomes necessary to distinguish key groupings in the data, perform statistics on these groupings, and then combine these statistics to infer overall effect. The statistic used for the individual groupings is identical to the Rayleigh’s z-statistic used in the first approach above, but applied separately to designated groupings of the data (in this case, individual tags). The statistic used to infer overall effect is a Hotelling’s F test of multiple comparisons. Individual vector plots of the difference between the swimming vector and the current vector were also created to address inter-individual behavior for each of the five different currents examined.

Since there were also potential issues with spatial and temporal autocorrelation, the second-order statistical analysis was also applied to groupings of the data defined by location and date. The overall spatial and temporal domain of tag data was divided into quartiles using a percentile methodology. These two sets of quartiles were then used to categorize the database into 16 separate groupings of approximately equal sample size spanning the entire domain. For this test the individual tags were aggregated if they overlapped in any of the 16 groupings to avoid low sample sizes and excessive numbers of groupings further defined by tag identity. For both applications of the second-order statistical analysis, the significance of the Hotelling’s F test as well as the mean of the second-order mean vectors were examined for departures from uniformity using the difference between the swimming vector and the current vector.

For directional data warranting further investigation (e.g. swimming directions), generalized additive modeling (GAM) was used to delineate relationships of these u and v values to seasonal, spatial, and current-dependent components. The GAM analyses used the library *mgcv* in the statistical programming language R, using Gaussian link functions and smoothing splines. Since it is likely that swimming behavior could be related to location and date (for migratory, seasonal, or age-specific behaviors), a suite of logical predictors were input into the GAM analyses. A 2-dimensional surface of longitude and latitude was used for a spatial term, and a smoothing spline as a function of date was used for the temporal term. Degrees of freedom were constrained manually to alleviate potential overfitting. To test for the existence of rheotaxis, both GAM analyses for u and v were allowed to use the underlying current u and v, respectively, as an input to the GAM. If the GAM analysis found a significant contribution from the respective current component, conditioned on all other potential descriptors, then this could be an additional line of evidence for the existence of rheotaxis.

For all of the approaches described above, all 5 current fields were used in independent analyses as a means of testing the sensitivity of the results to the particular current strata assumed to represent the primary zone of turtle occupancy. In other discussions where similar results were obtained across all products, only the shallow HYCOM analysis is presented.

## Results

**Figure 1:**
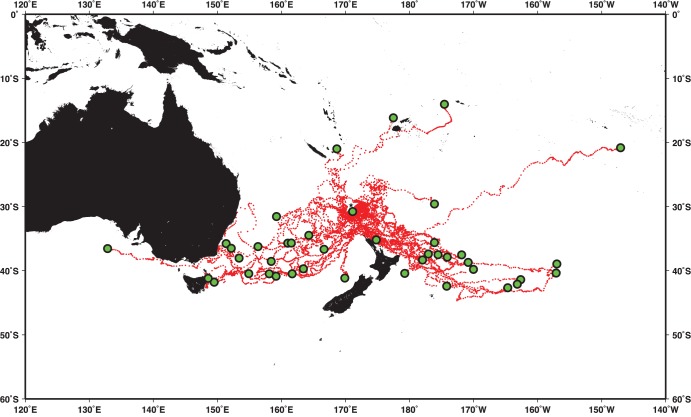
Satellite tag trajectories of 42 tagged oceanic juvenile loggerhead turtles. The single star denotes the release site for all 42 turtles, and the circles denote the final transmission site for each turtle.

The daily SSM satellite tag tracks are shown in Figure 1, with both the initial location and the location of final tag transmission sites indicated. The 42 turtles swam in a variety of directions with some tendency for either a southwest or southeast direction. However, several turtles ventured north, with one of the longest tracks to the northwest before transmission ceased in the vicinity of French Polynesia. One remarkable trajectory went to the southwest and passed through Bass Strait between Australia and Tasmania before transmission ceased. The final location of this tag was in offshore waters southwest of Port Lincoln, South Australia. A detailed summary of all tag movements is shown in Table 1. The number of transmission days ranged from 13–350 days with a median value of 178 days.

**Table 1:** Summary of satellite tag information for 42 juvenile loggerhead turtles released off New Caledonia on 9-September-2008.

ID Code	SCL (cm)	Weight (kg)	Final location	Date terminated	Distance traveled	Transmitting days
8552	28.5	4.7	43.78S 168.89W	12-Mar-2009	4,449 km	185
19597	26.0	4.1	29.60S 176.11W	14-Feb-2009	4,007 km	159
19599	29.1	5.1	37.39S 177.07W	20-Feb-2009	2,873 km	165
22151	28.6	4.6	40.37S 157.12W	9-Apr-2009	4,711 km	213
22168	29.2	5.4	37.51S 175.55W	18-Feb-2009	4,114 km	163
22270	32.3	7.2	41.79S 149.48E	12-Mar-2009	4,809 km	185
22275	27.1	4.6	35.63S 176.12W	25-Jan-2009	3,648 km	139
22980	28.5	4.9	16.16S 177.46E	18-Dec-2008	3,294 km	101
23465	25.7	4.2	40.50S 158.09E	28-Feb-2009	3,887 km	173
23483	25.6	3.6	42.66S 164.66W	7-Apr-2009	4,998 km	211
25313	27.6	5.2	38.71S 170.84W	8-Mar-2009	3,652 km	181
25359	29.5	5.2	41.41S 162.64W	10-Mar-2009	3,919 km	183
25695	27.4	4.5	34.49S 164.26E	16-Feb-2009	3,924 km	161
29060	27.3	4.4	14.05S 174.56W	12-Mar-2009	4,725 km	185
29067	24.7	3.4	35.70S 160.97E	6-Feb-2009	3,460 km	151
50134	28.8	4.6	36.24S 156.34E	4-Mar-2009	4,365 km	177
50137	25.5	3.7	36.66S 166.65E	7-Jan-2009	1,224 km	121
50143	29.1	5.2	20.82S 147.05W	31-May-2009	6,663 km	265
50145	24.0	3.2	42.42S 174.19W	12-Feb-2009	3,228 km	157
50147	29.4	5.5	38.34S 177.97W	23-Dec-2008	2,884 km	106
50148	32.0	7	20.99S 168.65E	30-Nov-2008	1,907 km	83
50149	26.5	4.3	38.55S 158.40E	12-Mar-2009	3,665 km	185
50150	27.4	4.2	35.69S 161.53E	20-Jan-2009	3,483 km	134
53744	27.9	4.9	35.21S 174.81E	26-Nov-2008	1,765 km	79
53747	29.7	6	38.05S 153.37E	24-Aug-2009	6,641 km	350
53748	28.4	5.5	35.78S 151.36E	10-Jun-2009	7,005 km	275
53752	24.9	3.6	36.52S 152.15E	29-May-2009	6,372 km	263
53754	28.6	5.4	40.86S 159.15E	6-Mar-2009	3,611 km	179
53757	27.0	3.8	36.55S 132.85E	23-Apr-2009	5,514 km	227
53758	28.3	4.5	39.80S 170.02W	18-Feb-2009	3,710 km	163
53759	29.2	5.2	31.55S 159.23E	22-Feb-2009	4,139 km	167
53762	27.8	5.2	40.50S 161.66E	26-Feb-2009	3,590 km	171
53763	25.9	3.8	37.53S 171.86W	6-Mar-2009	3,203 km	179
53765	29.7	6.1	37.88S 174.11W	7-Jan-2009	2,818 km	121
53766	31.0	6.1	41.15S 169.88E	10-Mar-2009	3,347 km	183
53767	28.3	4.6	38.95S 157.00W	6-Jun-2009	5,467 km	271
53769	25.9	3.6	39.71S 163.42E	3-Apr-2009	3,481 km	207
53770	28.2	5	30.78S 171.12E	21-Sep-2008	251 km	13
53771	27.5	4.1	40.47S 154.88E	5-Apr-2009	4,321 km	209
57144	26.0	4	41.21S 148.55E	12-Mar-2009	4,198 km	185
57151	34.3	8.1	42.11S 163.17W	20-Mar-2009	4,116 km	193
57152	26.3	4	40.42S 179.25E	18-Feb-2009	4,166 km	163

Argos ID code, turtle size (SCL, straight carapace length) and weight, final location, final date, distance traveled, and transmitting days are presented for each tagged turtle.

**Figure 2:**
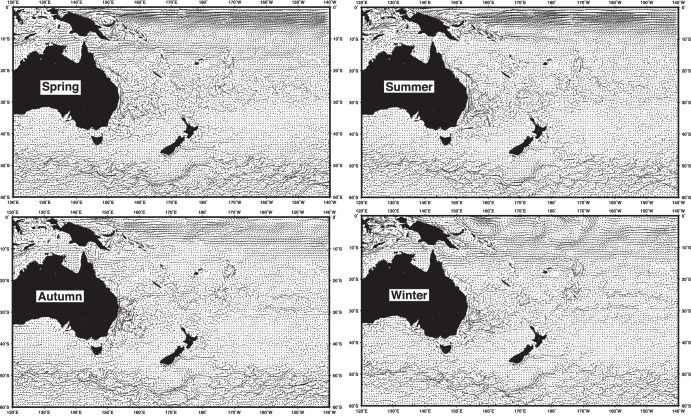
Seasonal HYCOM shallow currents (2008–2009 cycle: Spring = September–November, Summer = December–February, Autumn = March–May, and Winter = June–August.

Seasonal patterns of HYCOM shallow current flow over the time and space domain of this study are shown in Figure 2. The primary oceanographic features of the region appear to be adequately captured by the HYCOM data including, for example, the summer intensification of the East Australia Current (EAC) running southward along the eastern coast of Australia, the field of numerous mesoscale eddies in the Tasman Front region located off Eastern Australia extending to north of New Zealand, and the primarily winter Zeehan Current flowing to the southeast near Bass Strait and Tasmania. Current fields from the NOAA drifter buoys and OSCAR data were qualitatively similar to the surface HYCOM fields shown in Figure 2, and similarly for other HYCOM layers (intermediate and deep). It is difficult to detect vector differences visually over such a large scale map; therefore, other current fields are not shown as figures. It should be noted that the primary analysis of this study involved finer level comparisons in both space and time through examination of the resultant daily swimming vectors. Statistical evaluation of the different representations of current fields was not within the scope of this paper, but should be examined in greater detail in the future. Since small changes in currents (e.g., shear, Ekman dynamics) may be critically important for a swimming or drifting organism, this analysis used a broad array of current fields, which also served as a means of addressing sensitivity to changes in vertical stratification and diving behavior.

**Figure 3:**
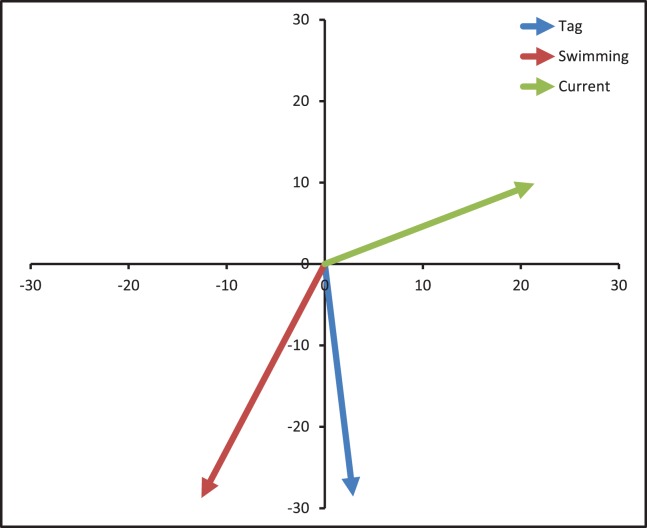
Average direction of tag movement, HYCOM shallow currents, and estimated swimming direction over the duration of this study. Axis units are in centimeters per second.

Over the duration of this study, the currents encountered by the oceanic juveniles were characterized similarly by all 5 measures for both speed and direction (Table 2). The average current speed encountered by the tagged turtles was estimated to be 21.37 cm/sec, averaged over the 5 current measurements examined (ranging from 14.55–28.62 cm/sec). Tag movement speed averaged 28.70 cm/sec. Swimming speed averaged 30.01 cm/sec (ranging from 25.46–32.94 cm/sec). The average current direction was to the North-Northeast, the average tag movement direction was to the South-Southeast, and the average swimming direction was to the South-Southwest (Figure 3).

**Table 2:** Summary of velocity and direction for movements by Tag, Current_D_, Current_O_, Current_HS_, Current_HI_, Current_HD_, Swimming_D_, Swimming_O_, Swimming_HS_, Swimming_HI_, Swimming_HD_, and Swimming_AVG_.

Entity	Average velocity (cm/sec)	Average direction (degrees)
Tag	28.70	174.22
Current_D_	28.62	91.64
Current_O_	14.55	66.50
Current_HS_	23.56	65.19
Current_HI_	21.37	64.04
Current_HD_	18.77	67.10
Current_AVG_	21.37	70.89
Swimming_D_	32.94	222.50
Swimming_O_	25.46	191.20
Swimming_HS_	31.36	203.64
Swimming_HI_	30.81	203.14
Swimming_HD_	29.49	202.69
Swimming_AVG_	30.01	204.63

The subscripts D, O, HS, HI, HD, and AVG refer to currents or swimming components estimated under current fields originating from Drifters, OSCAR currents, HYCOM Shallow currents, HYCOM Intermediate currents, HYCOM Deep currents, and Average currents, respectively.

**Figure 4:**
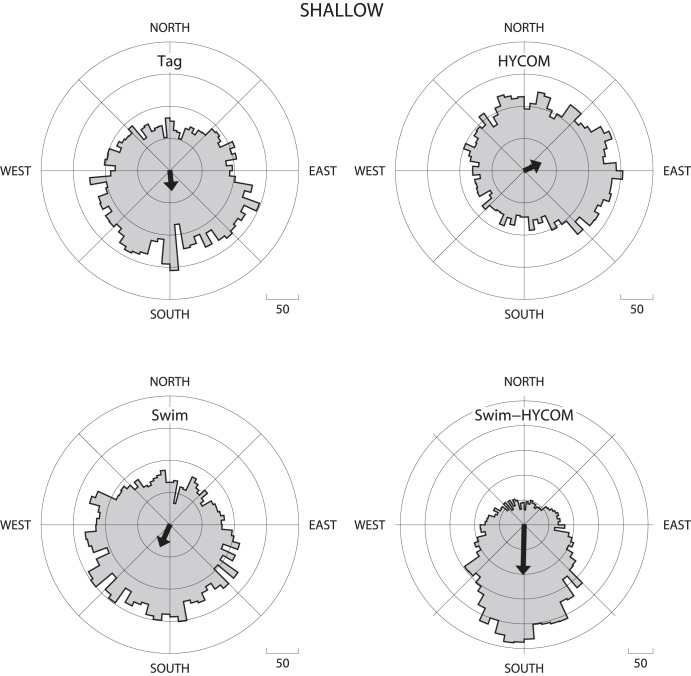
Tabulations of daily satellite tag displacement, HYCOM shallow currents, and inferred swimming direction. The swimming direction differencing to HYCOM shallow currents shown in the lower right panel is a relative direction where North is the direction of the HYCOM surface current and South is against the direction of the HYCOM surface current. Average unweighted resultant vector is plotted for each tabulation.

The tag movement direction, current direction, swimming direction, and the difference between the swimming and current directions are illustrated in Figure 4 as polar histogram distributions for the assumption of HYCOM shallow currents. The Rayleigh’s z test indicated that for all currents there were no significant departures from uniformity for the tag movement directions, the current directions, or the swimming directions. However, for drifters and all HYCOM layers there were highly significant departures from uniformity for the difference between the swimming direction and the HYCOM current direction (Table 3). When standardized to a polar orientation where north is the direction of the prevailing HYCOM current, all analyses indicated that the differences tabulated primarily in the relative southward direction, indicating that the swimming direction was against the HYCOM current (Figure 4).

**Table 3:** Summary of Rayleigh’s *z* statistical tests of directional uniformity applied to the daily directional data from tag movement, current direction, swimming direction, and the difference between the directions of swimming and prevailing current.

Current	Data tested	Rayleigh *z*	p	
Drifters	Tag	1.9551	0.14361	
	Current	1.5569	0.21738	
	Swimming	1.9327	0.14700	
	Swimming-Current	9.8637	0.00004	**
OSCAR	Tag	0.9714	0.39986	
	Current	0.3592	0.75628	
	Swimming	1.9462	0.14495	
	Swimming-Current	0.1126	0.97764	
HYCOM Shallow	Tag	0.9477	0.40983	
	Current	0.8379	0.45947	
	Swimming	1.4152	0.25192	
	Swimming-Current	6.1727	0.00178	**
HYCOM Intermediate	Tag	0.9477	0.40983	
	Current	0.8614	0.44835	
	Swimming	1.3676	0.26471	
	Swimming-Current	5.1644	0.00508	**
HYCOM Deep	Tag	0.9526	0.40778	
	Current	0.8685	0.44509	
	Swimming	1.3823	0.26069	
	Swimming-Current	3.1831	0.03999	*

The p values indicated by * and ** are indicative of statistically significant (p<0.05, p<0.01, respectively) directional departures from uniformity.

**Figure 5:**
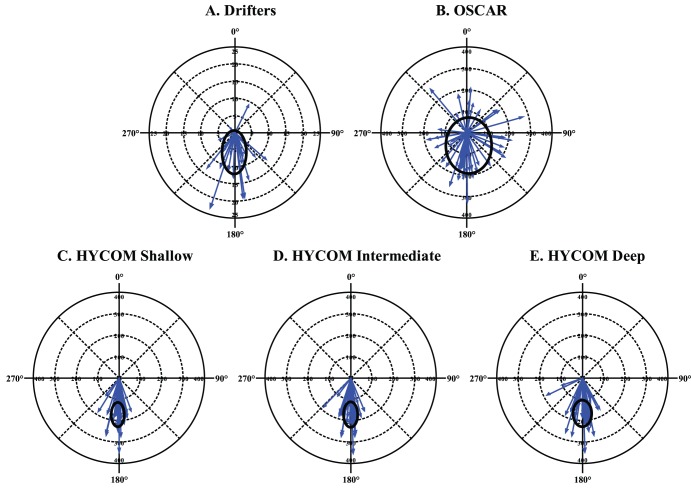
Individual vector representation of all 42 satellite tagged turtle movement showing the difference between the swimming vector and the current vector for each of the 5 currents examined. Each vector represents the average difference for that individual with the length of the vector proportional to the number of days at liberty for each individual. The swimming direction differencing to currents is a relative direction where North is the direction of the current and South is against the direction of the current. The standard deviation ellipse is also plotted, which represents the resultant vector per panel as a centroid of an ellipse with dimensions of standard deviations of direction differences and days at liberty.

The second-order statistical analyses were entirely consistent with the ad-hoc adjusted degrees of freedom approach. Analyses of all currents except OSCAR displayed evidence of non-uniformity for the difference between the swimming vector and the current vector (Table 4). All group means were clustered about the direction opposing the prevailing current direction. Across the 42 tags the mean of the second-order means was nearly exactly opposed to the prevailing shallow HYCOM current direction at 180.80 degrees with all tags displaying a similar value (95% C.I. = 177.32–184.39). Individual tags behaved similarly, with respect to the difference between swimming vector and current vector, across all currents with the exception of OSCAR (Figure 5). The second-order statistical analysis for spatial and temporal autocorrelation also showed a similar pattern against the prevailing shallow HYCOM current direction with a mean of the second-order means across all 16 spatial and temporal strata of 184.80 degrees with all strata displaying a similar value (95% C.I. = 175.20–196.39).

**Table 4:** Summary of Batschelet second-order statistical analysis of directional uniformity applied to the daily difference between the directions of swimming and prevailing current.

Current	Hotelling’s F	p		Grand mean (degrees)
Drifters	23.05	7.35E-07	**	180.04
OSCAR	2.88	0.0680		188.61
HYCOM Shallow	247.13	<1E-12	**	180.80
HYCOM Intermediate	224.00	<1E-12	**	179.51
HYCOM Deep	130.83	<1E-12	**	178.77

The p values indicated by * and ** are indicative of statistically significant (p<0.05, p<0.01, respectively) directional departures from uniformity.

**Figure 6:**
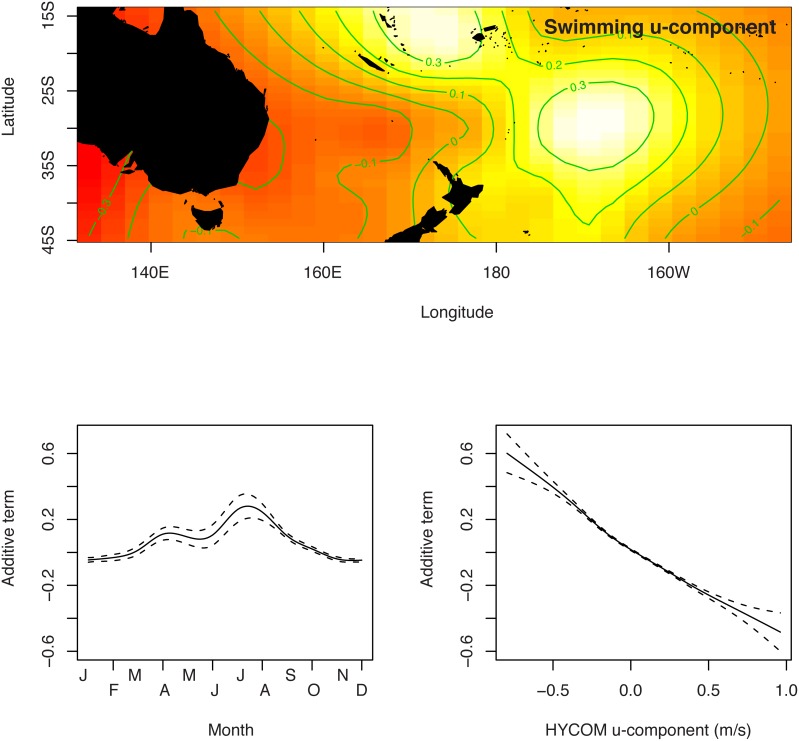
Generalized additive model for u-component (east/west) of oceanic juvenile loggerhead turtle swimming direction estimated from HYCOM shallow currents.

**Figure 7:**
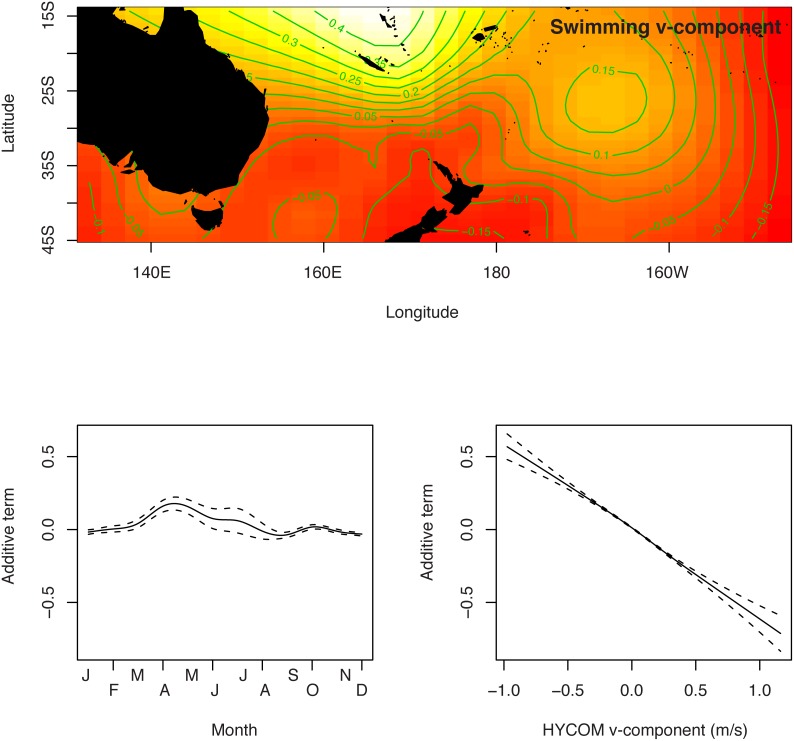
Generalized additive model for v-component (north/south) of oceanic juvenile loggerhead turtle swimming direction estimated from HYCOM shallow currents.

The absolute swimming direction was decomposed into an East/West u-component and a North/South v-component. These u-component and v-component from the HYCOM shallow analysis were fit to GAMs with a spatial term, a seasonal term, and a corresponding current term (u-component or v-component, depending on the swimming component being fit). All terms were highly significant and further tuning of the GAM models were unnecessary, aside from adjustments to degrees of freedom to avoid overfitting (determined visually when the GAM smoother functions had excessive inflection points). The spatial term for the swimming u-component suggested that the swimming direction was both a function of longitude and latitude with continued westward swimming predicted in the west, and continued eastward swimming in the east, with a zone of high eastward swimming centered at 30S and 165W (Figure 6). The spatial term for the swimming v-component suggested that swimming direction was both a function of longitude and latitude with continued northward swimming to the north, and generally southward swimming continuing to the south and to the east (Figure 7). There was a peak in eastward swimming in July–August and a slight peak in northward swimming in April–May. The relationship between swimming u-component and swimming v-component was negative and nearly linear despite the GAM smoothing spline functions being capable of curvilinear additive functions with variable degrees of freedom. The spatial and temporal terms for the intermediate and deep HYCOM GAM analyses were qualitatively similar to the shallow HYCOM GAM analysis, differing primarily in magnitude of the estimated swimming values since the surface layer tends to move more quickly than deeper layers and, therefore, has an associated faster estimated swimming speed to reconcile with the same net tag displacement.

## Discussion

The polar histograms of the difference between the swimming directions and the prevailing current directions, coupled with the negatively linear GAM terms for swimming u-component and v-component as a function of the corresponding u-component and v-component, are strong evidence of positive rheotaxis occurring in an oceanic organism. There are fewer confounding cues available to an oceanic organism than to the same organism in an experimental laboratory apparatus where presumed rheotactic responses could be simpler orientation to an odor gradient, temperature gradient, or aided by the fixed orientation of the apparatus. For example, if the organism is positioned on the substrate or has a visual fix on the substrate, and wishes to respond to a current, it is a simple matter to re-orient towards the current given the tactile or visual cues of the surrounding environment. However, for an organism in a homogenous and moving liquid environment, both the detection and re-orientation towards the current can be much more complicated. The data and analyses presented here propose that oceanic juvenile loggerhead turtles have such a capability.

It is noteworthy that the comparisons using OSCAR currents were not significant for showing a rheotactic response. While this might be compelling evidence to counter the proposed finding, we feel the most likely explanation for the weaker patterns in the OSCAR analysis is that it is the only ocean current product not providing a daily estimate, as OSCAR is a 5-day composite dataset due to the geometry of satellite orbital cycles and altimeter/scatterometer swath widths. It is difficult to measure animal behavior cued to the environment if the environmental measure is an average over 5 days, while the behavior is measured over the course of a single day. Given the daily NOAA drifter buoy analysis showed evidence of rheotaxis, and that the OSCAR data is essentially tuned to NOAA drifter buoy movement, we feel the lack of significant finding in the OSCAR analysis does not in any way preclude existence of positive rheotaxis in oceanic juvenile loggerhead turtles. The consistent finding in all 3 strata of HYCOM currents (with the HYCOM shallow arguably the most suitable estimate) remains strong evidence of positive rheotaxis.

A similar study on green and leatherback turtles did not find any evidence of non-random directional swimming with respect to the prevailing current [33], [34]. It should be noted however that these studies on green and leatherback turtles used a current product very similar to the OSCAR data (composite of geostrophic and wind-driven components) and is of a relatively coarse resolution in both space and time due to the satellite track geometry in comparison to HYCOM data. Again, it may be difficult to infer animal behavior from a product that is spanning over multiple days in its estimation and expression. Geostrophic flow fields are based on satellite altimeter sensors with orbit repeat cycles of typically 10 days. While wind data from scatterometer sensors can be daily, the final merged product still requires much smoothing and extrapolation for a daily estimate of flow at any given location. Additional flow fields should be examined, particularly modeled flow fields which can better match the tagged animal data resolution, or flow fields generated from simple drifter buoys appear capable of capturing the rheotaxic effect. Perhaps unfortunately, several other turtle studies examining pelagic movement and oceanography have also relied on coarse resolution merged geostrophic and wind-stress products [35], [36].

The vector geometry approach used in this analysis could also be interpreted with respect to mechanisms other than active upstream-oriented swimming. In fact, the findings do not indicate that oceanic juvenile loggerhead turtles are swimming upstream as a migrating salmon would, for example, countering and overcoming an encountered flow field. The data indicate the transport is resisted yet not necessarily overcome in all instances, based on the vector directions and magnitudes. The turtles may be able to resist advection by some means such as a person swimming in a river might backpedal or gently scull with one’s hands when entrained in the main flow field if attempting to maintain position as cued by visual fixes on the shoreline or river bottom. If the turtle was simply attempting to stay in the same location while knowingly being advected, the resultant behavior to maintain that position would manifest as a swimming vector against the background current flow such as documented here. Knowledge of body orientation would be helpful towards resolving this question. Body orientation with respect to prevailing current direction, swimming direction, and net displacement direction would be very insightful towards understanding what the individual is attempting to do.

The resultant swimming vector against the current flow could also be interpreted to be caused by frictional drag slowdown of an object in a flow field, or what is often referred to as slippage. Slippage can account for the difference between a predicted trajectory and an actual trajectory if the object is moving slower than the ambient flow field due to frictional drag on the object. Slippage estimates of surface-tethered drifter buoys have indicated that slippage can be on the order of 13% of the existing current [37], which for this particular situation would predict slippage of 2.8 cm/sec (given an average current of 21.37 cm/sec). Other researchers have indicated that drifter buoy slippage can be 2 cm/sec [38] or 4.5–9 cm/sec [39]. None of these estimates of slippage come close to the estimated 30 cm/sec swimming vector of this study. Hence, attributing the rheotactic response to simple frictional slippage is quite unlikely. The swimming magnitude is approximately 10 times any known slippage factor. Furthermore, the buoy estimated slippage is also accounting for the tether line and a surface buoy, which the sea turtles do not have. Slippage of a simple object would be substantially less than the 2–9 cm/sec estimates from tethered drifter buoys.

Frictional drag of the satellite tag could be similar to the slippage process, whereby this and potential windage on the emergent tag and/or carapace could serve as a mechanism for disrupting active swimming and/or imposing differential drift from surface currents [40]. Drag estimates for the species, carapace length, and tags in this study would be on the order of a 20–22% increase in drag, not including windage effects for sustained surface intervals. However, since the sea turtles appear to be swimming against the current, the estimated rheotaxis is likely conservative, i.e., in the absence of tag drag the magnitude of the swimming response may be higher than what is presented here. Nevertheless tag drag should be addressed in all tagging studies involving attachment of tags to mobile animals, particularly if the size of the tag is large relative to the body size of the organism. Size, weight, buoyancy should be carefully considered [41].

It remains speculative to identify the mechanism for detecting a current while being moved in that same current. However, directional cues might be inferred by traversing through a region of current shear or by using a similar processing of differential movement at the air-sea interface. This would require knowledge about the state of motion in the adjacent layer, which may be difficult. Wave action might be useful as an indicator of current direction. While the interplay of wind, waves, and currents is a complex process, it remains plausible that an organism could glean some measure of directional movement by the nature of the prevailing wave action [15]. Wind driven currents would likely be the simplest to estimate, since there should be a strong differential in the speed of the wind and the speed of the water, with a nearly constant offset due to the Coriolis effect. Oscillatory movement in the water column might also be useful to infer direction of travel, if wave action and current flow are tightly coupled, as in the case of wind-driven surface currents. Particulate matter in the water could be used as a visual fix to facilitate this orientation. The utility of visual cues could be experimentally examined with existing data to examine the rheotactic patterns separated by day and night with attention to moon phase. If visual cues to interfaces, waves, particulate matter, etc. are critical, then this pattern should break down on dark nights. It is also conceivable that there may be a multiple cues utilized, particularly if a wide range of oceanographic conditions are encountered and if there has been evolution of a long-distance migration route [42].

Another potential mechanism is that an object being pushed by water currents will experience differential pressure on one side of its body versus the opposing side. An application of this is seen in the rheotactic responses experimentally examined using artificial intelligence. In this example, a positively rheotactic fish robot was successfully designed and tested using 2 pressure sensors to determine orientation with respect to a current flow [43]. Such a design is very analogous to the mechanosensory receptors on a lateral line for example, which is found on fish and some amphibians. Nerves embedded in the sea turtle carapace may be able to function in this fashion, though this has yet to be documented. Electronic sensors and circuitry are a poor substitute for living organisms and their neurological capabilities. Hence, it would seem quite likely that simple rheotactic responses would have evolved if this type of response were adaptive for the species. While this artificial intelligence response has been shown to occur in a narrow flow tunnel, it is mechanistically identical to an oceanic condition where a flow field is pushing (or pulling) at an object of different (higher) density and not simply being carried along with it in uniform fluidic unity. Furthermore, flow fields in the ocean do change over time and space, and the differential response of different masses and densities of objects therein might allow for a simple directional fix on the advective movement using changes in pressure.

The adaptive value of rheotaxis can exist if there is benefit to swimming upstream. At its most rudimentary form, this could be a means to minimize dispersive losses to an organism that relies on land. This would allow an organism and progeny to remain in the vicinity of the optimal habitat chosen to reproduce in. Swimming against an advective flow in this situation would be adaptive if mortality was high due to insufficient habitat in other portions of the aquatic system. Generally, there are two responses by an organism when it is pushed: it can either retreat or push back. In this instance the taxis appears to be positive, in that once detected, the reaction is to swim towards the stimulus. It would be extremely useful to have knowledge of body orientation to determine if the turtle is swimming headlong into the flow or being carried with the flow, yet arresting its advection by some means. Directional sensors and/or video cameras should be incorporated into future telemetry studies.

If the flows were predominantly meridional in a particular area then it would be advantageous to resist advection since transport to either lower latitudes or higher latitudes would likely be deleterious for organisms preferring a certain temperature range or with other environmental constraints. While flow fields are difficult to generalize over time and space, the location of this study area is in the western portion of the South Pacific Gyre, a large water mass generally spinning in a counter-clockwise direction below the equator between Australia and South America. Ectothermic organisms such as sea turtles would likely be more concerned with avoiding cold water than warm water and, hence, a swimming response against the southward current might manifest itself as adaptive behavior. The importance of temperature for loggerhead habitat has been well studied in the North Pacific Ocean [22], [44], [45]. Additional study in the South Pacific Ocean is clearly needed.

Several other studies are in progress using data from this particular tagging experiment. The swimming u and v estimated from this study will be utilized in a computer simulation intended to mimic the movement of oceanic juvenile loggerhead turtles from a similar point-source release (Kobayashi et al., in review). Additionally, a wider suite of environmental variables have been examined and will be used to characterize oceanic juvenile habitat in the South Pacific, similar to what was accomplished in the North Pacific [22].

In conclusion, the significant findings of this study include the identification of a positive rheotaxis response in a pelagic organism. This finding will aid in our understanding of oceanic movements of juvenile marine turtles. This paper is another example of active orientation including likely multiple sensory cues that allow juvenile turtles to orient and offset passive displacement due simply to wind and ocean currents.
