# Correction: “Going with the Flow” or Not: Evidence of Positive Rheotaxis in Oceanic Juvenile Loggerhead Turtles (*Caretta caretta*) in the South Pacific Ocean Using Satellite Tags and Ocean Circulation Data

**DOI:** 10.1371/journal.pone.0111358

**Published:** 2014-10-13

**Authors:** The PLOS ONE Staff

There is a missing funder in the Funding section. Please refer to the complete funding statement here:

We thank Karen Frutchey and Raymond Clarke of the Pacific Islands Regional Office, National Marine Fisheries Service (NMFS) and National Oceanic and Atmospheric Administration (NOAA) who provided key funding towards this project. The funders were base funding and add-on funding from the United States government to NOAA and had no role in study design, data collection and analysis, decision to publish, or preparation of the manuscript.
